# Targeting the spliceosome for cutaneous squamous cell carcinoma therapy: a role for c-MYC and wild-type p53 in determining the degree of tumour selectivity

**DOI:** 10.18632/oncotarget.25196

**Published:** 2018-05-01

**Authors:** Lydia A. Hepburn, Angela McHugh, Kenneth Fernandes, Garry Boag, Charlotte M. Proby, Irene M. Leigh, Mark K. Saville

**Affiliations:** ^1^ Division of Cancer Research, School of Medicine, University of Dundee, Dundee DD1 9SY, UK; ^2^ Centre for Cutaneous Research, Barts and The London School of Medicine and Dentistry, Queen Mary University of London, London E1 2AT, UK

**Keywords:** squamous cell carcinoma, spliceosome, c-MYC, p53, MDM2

## Abstract

We show that suppression of the spliceosome has potential for the treatment of cutaneous squamous cell carcinoma (cSCC). The small-molecule inhibitors of the spliceosome at the most advanced stage of development target the splicing factor SF3B1/SF3b155. The majority of cSCC cell lines are more sensitive than normal skin cells to death induced by the SF3B1 inhibitor pladienolide B. Knockdown of SF3B1 and a range of other splicing factors with diverse roles in the spliceosome can also selectively kill cSCC cells. We demonstrate that endogenous c-MYC participates in conferring sensitivity to spliceosome inhibition. c-MYC expression is elevated in cSCC lines and its knockdown reduces alterations in mRNA splicing and attenuates cell death caused by interference with the spliceosome. In addition, this study provides further support for a key role of the p53 pathway in the response to spliceosome disruption. SF3B1 inhibition causes wild-type p53 upregulation associated with altered mRNA splicing and reduced protein expression of both principal p53 negative regulators MDMX/MDM4 and MDM2. We observed that wild-type p53 can promote pladienolide B-induced death in tumour cells. However, p53 is commonly inactivated by mutation in cSCCs and p53 participates in killing normal skin cells at high concentrations of pladienolide B. This may limit the therapeutic window of SF3B1 inhibitors for cSCC. We provide evidence that, while suppression of SF3B1 has promise for treating cSCCs with mutant p53, inhibitors which target the spliceosome through SF3B1-independent mechanisms could have greater cSCC selectivity as a consequence of reduced p53 upregulation in normal cells.

## INTRODUCTION

cSCC is responsible for a substantial proportion of deaths from skin cancer and can also have a considerable impact on the quality of life [1–5]. Immunosuppressed patients, including organ transplant recipients, have an increased incidence of aggressive cSCCs [2]. In patients with the genetic skin blistering disease severe generalized recessive dystrophic epidermolysis bullosa (RDEB) the cumulative mortality risk from cSCC is 80% by the age of 55 [4]. There is a need for improvements in the treatment of cSCC both in the general population and in high-risk groups [1–7]. Effective systemically-delivered therapy is required for cSCC patients with distant metastasis. In addition, some cSCC patients with localised disease would benefit from improved directly-delivered therapy (topical treatment or intratumoral injection) [1, 7–9].

Our siRNA screen identified the spliceosome as a potential target for cSCC therapy. The spliceosome generates mature mRNA by the splicing of pre-mRNA which involves the removal of introns and the joining of exons. This is essential for gene expression. In addition, alternative splicing makes a major contribution to genetic diversity. The spliceosome contains over one hundred and fifty different proteins (splicing factors) and its structure and composition are highly dynamic [10, 11]. Core components of the spliceosome are the U1, U2, U4, U5 and U6 small nuclear ribonucleoprotein (snRNP) particles [12–14]. Each snRNP contains a small nuclear RNA (snRNA) and the spliceosome is thought to be a ribozyme where the snRNA play a catalytic role that is directed by associated proteins [11, 15]. *In vitro* studies show that the U1 snRNP interacts with the 5′ splice site and the U2 snRNP associates with the intronic branch-point. This is followed by the recruitment of the U4/U6.U5 tri-snRNP. The U1 and U4 snRNPs are destabilised and the spliceosome catalyses two transesterification reactions. A bond is formed between the 5′ splice site and an adenosine in the branch-point causing cutting of the intron and this is followed by ligation of 5′ and 3′ splice sites.

There is growing interest in targeting the spliceosome for cancer therapy [16–18]. The spliceosome may appear to be a surprising therapeutic target because of its importance in normal cells. However, cancers can be more susceptible than untransformed cells to spliceosome inhibition [19–21]. Importantly, only a subset of splicing events is affected by knockdown of a particular core splicing factor: there are alterations in splice site selection rather than generalised inhibition of splicing and the effects of suppressing different core splicing factors can be divergent [22]. In support of the ability of patients to tolerate spliceosome inhibition many therapies which are commonly used to treat cancer have affects on the spliceosome and pre-RNA splicing, including DNA damaging agents and 5-fluorouracil [23–25]. For example, 5-fluorouracil is incorporated into the U2 snRNA which interferes with splicing [23].

The most advanced small-molecule spliceosome inhibitors target the SF3B complex which is a multisubunit component of the U2 snRNP. SF3B binds to pre-mRNA in the vicinity of the branch-site and consequently participates in splice site recognition and selection [26]. Several families of naturally occurring compounds with anti-tumour activity have been found to target the spliceosome through an interaction with this complex [16, 18]. Synthetic analogues of these inhibitors have now been generated [21, 27, 28]. The splicing factor SF3B1 is one of seven subunits of the SF3B complex and it is thought to be a direct target for these compounds [29–31]. Pladienolide B is is an example of a naturally occurring spliceosome inhibitor that interacts with SF3B1 [32, 33]. A point mutation in SF3B1 has been shown to decrease the binding of pladienolide B to the spliceosome and to dramatically reduce the potency of its effects on cell viability [29]. SF3B1 inhibitors have good pre-clinical anti-tumour activity in model systems [17, 21, 32, 34, 35]. Systemically delivered E7107 was the first SF3B inhibitor to be tested in clinical trials but there were adverse effects in a small number of patients [36, 37]. The SF3B inhibitor H3B-8800 has recently entered a phase 1 clinical trial involving oral delivery for patients with haematological malignancies (NCT02841540). Additional small molecule modulators of the SF3B complex are candidates for testing in clinical trials [28].

A number of pathways can influence the sensitivity of cell viability to interference with the spliceosome. Ectopic expression of the transcription factor c-MYC sensitises normal cells including neural stem cells, fibroblasts and mammary epithelial cells, to modulation of the spliceosome [19, 38]. It has been proposed that c-MYC upregulation places a burden on the spliceosome by causing a widespread increase in transcription [19]. Synthetic lethality with spliceosome inhibition could provide a means to treat the many tumours with elevated c-MYC [39, 40]. This is of great interest because c-MYC is difficult to target directly [41, 42]. Altered splicing of the mRNAs coding for the anti-apoptotic proteins BCL-X and MCL-1 can also contribute to the anti-tumour activity of targeting the spliceosome [43–48]. In addition, we have shown previously that interference with the spliceosome by multiple mechanisms results in wild-type p53 activation [49]. Consistent with this three splicing factors were top ten hits, ranked according to the magnitude of p53 transcriptional activation, in a recent genome wide siRNA screen in non-small cell lung cancer cells [50]. In unstressed cells p53 is held in check by MDMX and MDM2 [51, 52]. They both inhibit the transcriptional activity of p53. MDM2 is an E3 ligase which promotes p53 ubiquitination leading to degradation of p53 by the proteasome. MDMX can stimulate the ubiquitin ligase activity of MDM2 by forming an MDMX/MDM2 heterodimer. We reported that a reduction in the level of MDMX acts as a sensor of alterations in the spliceosome independently of DNA-damage signalling [49]. Similar observations have now been made in other *in vitro* and *in vivo* systems [50, 53, 54]. This can involve changes in MDMX mRNA splicing due to weak splice sites that are sensitive to interference with the spliceosome but other mechanisms may also contribute, including enhanced MDMX protein degradation [49, 50, 53, 54].

In this study we observed that suppression of the spliceosome by targeting a range of splicing factors can selectively kill cSCC cells compared to normal skin cells. We sought to identify mechanisms that influence this tumour selectivity. Our results indicate that endogenous c-MYC upregulation contributes to sensitising cSCC cells to inhibition of the spliceosome. In addition, we show that wild-type p53 can play a dominant role in promoting cell death caused by small molecule SF3B1 inhibition in both tumour cells and in normal skin cells. Consistent with previous observations, suppression of SF3B1 reduces the expression of MDM2 as well as MDMX and causes strong upregulation of wild-type p53 [49, 55]. We show that small molecule inhibition of the splicing factor SF3B1 has promise for treating cSCCs with mutant p53. However, this study suggests that by avoiding robust p53 upregulation in normal cells SF3B1-independent suppression of the spliceosome could further extend the therapeutic window for cSCCs in which normal p53 function is lost.

## RESULTS

### Targeting the spliceosome can selectively kill cSCC cells

**Figure 1:**
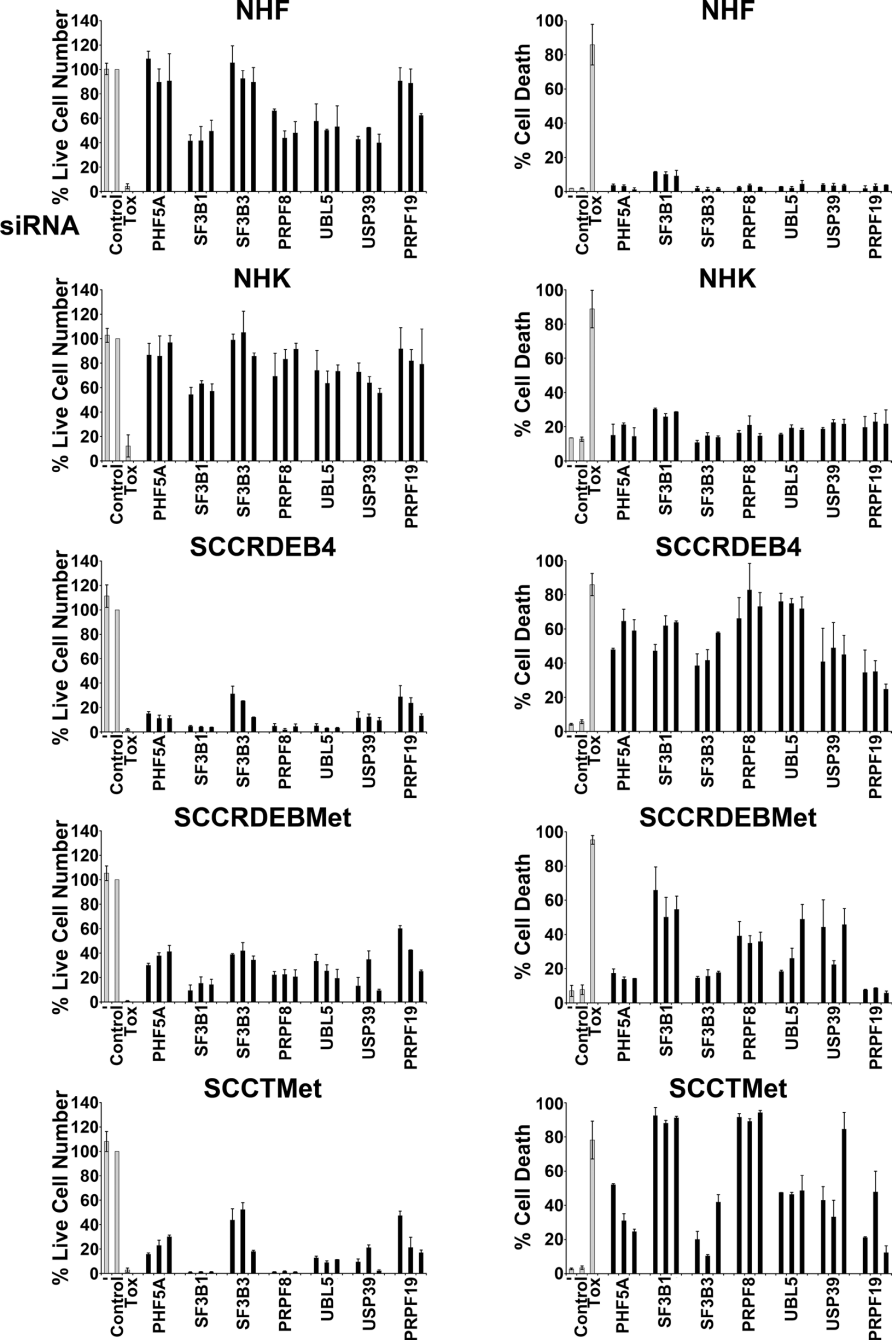
Splicing factor knockdown can selectively kill cSCC cells Normal skin cells (NHF, NHK) and cSCC lines derived from a primary tumour (SCCRDEB4) and a metastatic tumour (SCCRDEBMet) from RDEB patients and a metastatic tumour from a transplant recipient (SCCTMet) were mock transfected (−), transfected with a non-targeting siRNA (Control) or transfected with three individual siRNAs targeting the indicated splicing factors. A cytotoxic siRNA (TOX) was used as a positive control for transfection efficiency. Cell viability (live cell number) expressed as a percentage of carrier alone and the percentage of dead cells were assayed by real-time imaging 96 hours after transfection. The values are the mean −/+ range of two independent experiments. Splicing factor knockdown generally caused a larger reduction in viability and a greater level of cell death in cSCC cell lines than in normal skin cells.

To identify potential targets for cSCC therapy an siRNA screen was carried out in SCCRDEB4 cells with reduced cell viability as a readout (data not shown). Based on Cvitkovic et al., [10] the library used for the screen contained siRNAs targeting ten splicing factors [56, 57]. Six of these splicing factors: SF3B3/SF3b130, PHF5A/SF3b14b, PRPF8/PRP8, UBL5/HUB1, USP39 and PRPF19 were among the top hits. SF3B3 and PHF5A are components of the SF3B complex [26]. PRPF8 occupies a core position in the spliceosome and is involved in maintaining the activate site conformation and in promoting splicing reactions [11, 58]. UBL5 may play a role in both early and later stages of splicing through binding to the DEAD-box helicase PRPF5/DDX46 which is involved in spliceosome assembly and to SART1/Snu66 a component of the U4/U6.U5 tri-snRNP [59, 60]. USP39 contributes to the recruitment of the U4/U6.U5 complex to splice sites [61]. PRPF19 is part of the nineteen complex (NTC) which participates in activation of the spliceosome [62, 63]. We compared the effect of knockdown of these splicing factors on viability (live cell number) and death in normal human fibroblasts (NHF) and normal human keratinocytes (NHK) and cell lines derived from primary and metastatic cSCCs from RDEB patients (SCCRDEB4 and SCCRDEBMet) and a metastatic cSCC from a transplant patient (SCCTMet). The effect of knockdown of the SF3B subunit SF3B1 was also investigated because it is a target of spliceosome inhibitors [29–31]. The use of a positive control cytotoxic siRNA and analysis of splicing factor knockdown indicated high transfection efficiency in normal and tumour cells (Figure 1 and Supplementary Figure 1). Splicing factor knockdown caused a greater reduction in the viability of cSCC cell lines than normal skin cells (Figure 1, left panels). Strikingly, splicing factor depletion also generally caused a higher level of death in the cSCC cells (Figure 1 right panels). Knockdown of SF3B1, PRPF8, UBL5 and USP39 increased cell death in all cSCC cell lines. Suppression of the SF3B complex subunits PHF5A, SF3B1 and SF3B3 resulted in a similar high level of death in SCCRDEB4 cells. In contrast, depletion of SF3B1 was more effective in killing SCCRDEBMet and SCCTMet cells than knockdown of PHF5A or SF3B3. Suppression of PRPF19 killed SCCRDEB4 and SCCTMet cell lines but it did not increase cell death in SCCRDEBMet cells. These results indicate that splicing factor depletion can selectively kill cSCC cells derived from both primary and metastatic tumours but there are variations in the potency of the anti-cSCC activity of targeting different splicing factors.

**Figure 2:**
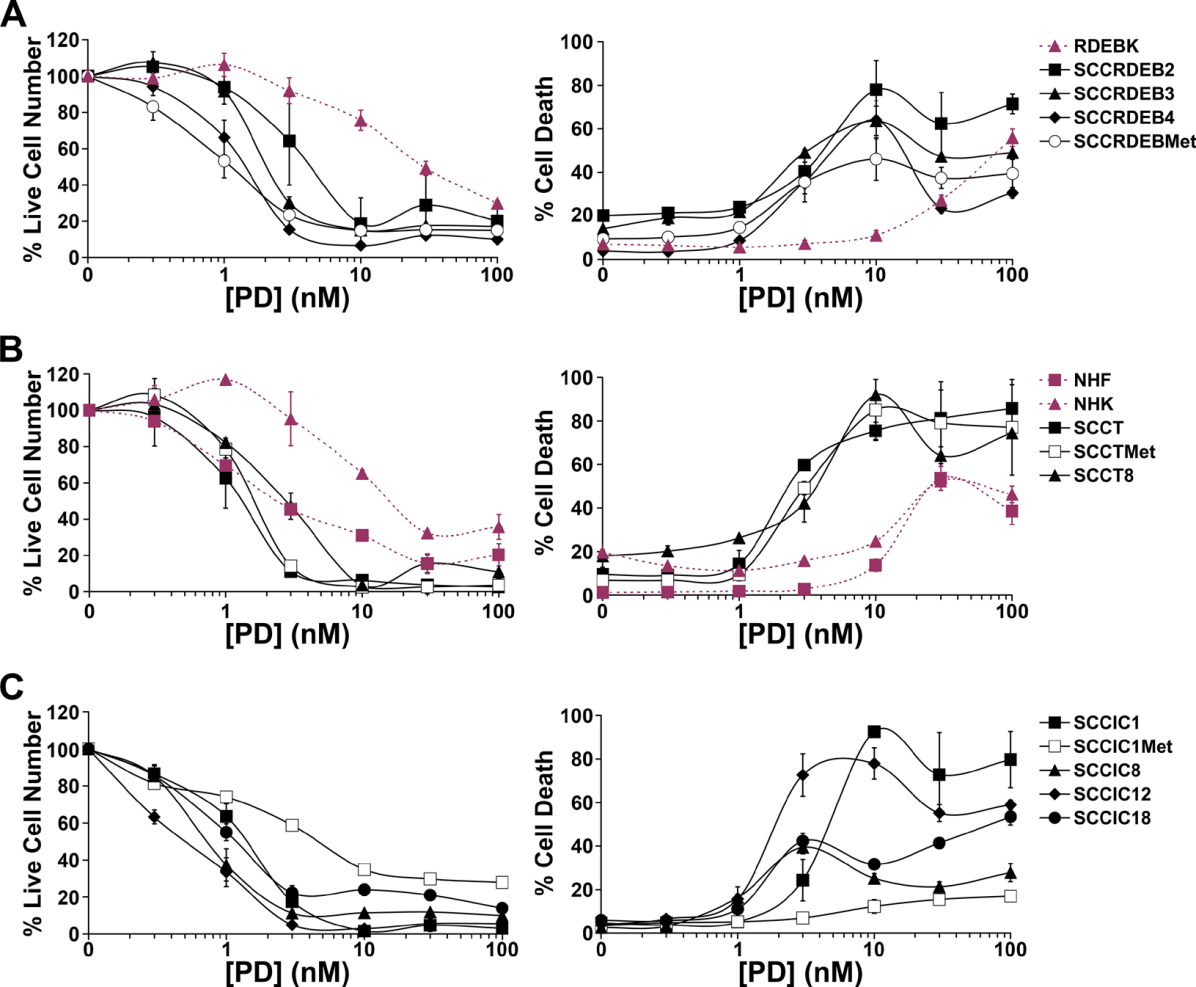
The small-molecule SF3B1 inhibitor pladienolide B selectively kills cSCC cell lines Normal skin cells and cSCC cell lines were incubated with the indicated concentration of pladienolide B (PD) for 72 hours. Cell viability (live cell number) expressed as a percentage of carrier alone and the percentage of dead cells were assayed by real-time imaging. Values are the mean −/+ SEM of at least three independent experiments (NHF, RDEBK, SCCRDEB4, SCCRDEBMet, SCCIC8, SCCIC12, SCCIC18) or the mean −/+ range of two independent experiments (NHK, SCCRDEB2, SCCRDEB3, SCCT, SCCTMet, SCCT8, SCCIC1, SCCIC1Met). (**A**) Keratinocytes (RDEBK) and cSCC cell lines derived from RDEB patients (SCCRDEB). (**B**) NHF and NHK. cSCC cell lines derived from transplant patients (SCCT). (**C**) cSCC cell lines derived from immunocompetent patients (SCCIC) The majority of cSCC cell lines were killed at lower concentrations of pladienolide B than normal skin cells.

The effect of the small molecule SF3B1 inhibitor pladienolide B on cell viability and death was determined in normal skin cells and twelve cSCC cell lines. cSCC cell viability was generally more pladienolide B-sensitive than that of NHK and normal keratinocytes from an RDEB patient (RDEBK) (Figure 2, left panels). The viability of NHF was partially reduced at low pladienolide B concentrations due to inhibition of proliferation and further inhibited at high concentrations associated with increased cell death (Figure 2B). Importantly, most cSCC cell lines were killed at lower concentrations of pladienolide B than required to kill normal skin cells, including all cSCC lines derived from RDEB and transplant patients (Figure 2, right panels). SCCIC1Met, SCCIC8 and SCCIC18 cells were the least sensitive to pladienolide B-induced death. In some instances the proportion of dead cells declined at higher pladienolide B concentrations. This may reflect a shift in the balance of pro and anti-death pathways due to variations in the degree of inhibition of splicing events at different pladienolide B concentrations. Overall these results indicate that small-molecule inhibition of SF3B1 has potential for cSCC therapy.

### c-MYC is a determinant of cSCC sensitivity to targeting the spliceosome

**Figure 3:**
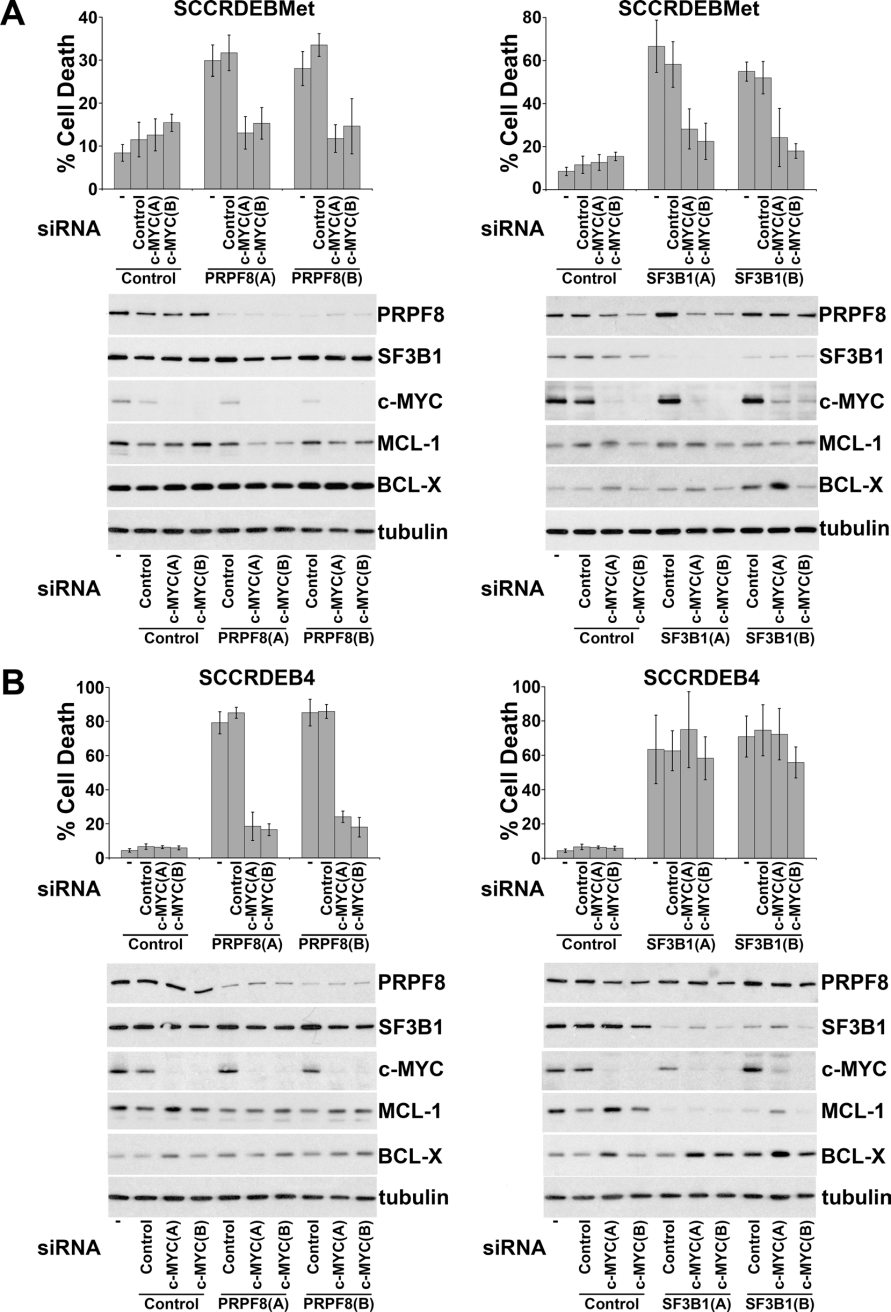
Endogenous c-MYC can confer sensitivity to PRPF8 and SF3B1 knockdown in cSCC cells Cells were mock transfected (−) or transfected with the indicated combinations of non-targeting siRNA (Control) and two individual siRNAs: (A) and (B) targeting c-Myc, PRPF8 and SF3B1. The percentage of dead cells was determined by real-time imaging. The values are the mean −/+ SD of three independent experiments. Expression of the indicated proteins was analysed by western blotting. (**A**) SCCRDEBMet cells. (**B**) SCCRDEB4 cells. c-MYC depletion attenuated cell death resulting from splicing factor knockdown in all cases except for SF3B1 knockdown in SCCRDEB4 cells. Splicing factor knockdown had little effect on full-length MCL-1 or BCL-X protein expression with the exception of SF3B1 knockdown in SCCRDEB4 cells which reduced MCL-1 levels.

**Figure 4:**
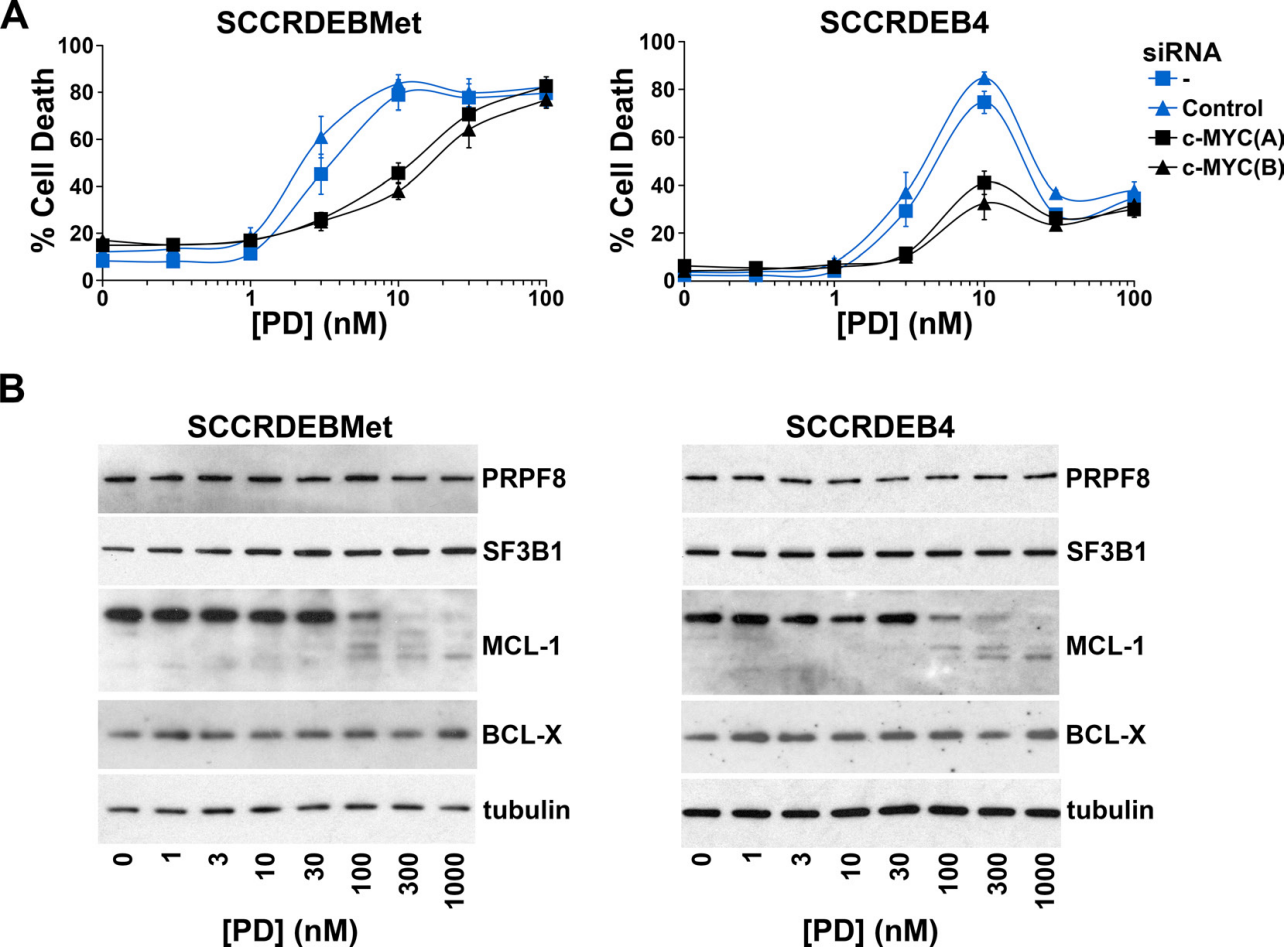
In cSCC cells endogenous c-MYC confers sensitivity to the SF3B1 inhibitor pladienolide B (**A**) SCCRDEBMet and SCCRDEB4 cells were mock transfected (-) or transfected with the indicated siRNAs 24 hours prior to incubation with pladienolide B (PD). The percentage of dead cells was determined by real-time imaging. Values are the mean −/+ SEM of three independent experiments. Knockdown of c-MYC increased resistance to pladienolide B-induced death. (**B**) SCCRDEBMet and SCCRDEB4 cells were treated with pladienolide B (PD) and protein expression was analysed by western blotting. Full-length MCL-1 expression was reduced only at a relatively high pladienolide B concentration which was more than 10-fold greater than required to kill cSCC cells and at which cell death was not affected by c-MYC depletion.

Ectopic expression of c-MYC in normal cells enhances sensitivity to spliceosome inhibition [19, 38]. Alterations in mRNA splicing of BCL-2 family members can promote cell death brought about by targeting the spliceosome [43–48]. We investigated the involvement of c-MYC and regulation of BCL-2 family members in spliceosome-suppression induced death in cSCC cells. SF3B1 was selected for further study because of its importance as a small-molecule target [20, 29, 31]. PRPF8 was also selected to investigate whether common mechanisms may be involved in determining sensitivity to different ways of targeting the spliceosome. Knockdown of c-MYC attenuated cell death caused by depletion of PRPF8 or SF3B1 in SCCRDEBMet cells and depletion of PRPF8 in SCCRDEB4 cells (Figure 3). In contrast, knockdown of c-MYC had no effect on cell death resulting from depletion of SF3B1 in the SCCRDEB4 cell line (Figure 3B). SCCRDEBMet and SCCRDEB4 cells express MCL-1 and BCL-X but relatively low levels of BCL-2 (Supplementary Figure 2). There was little or no effect of PRPF8 or SF3B1 knockdown on the level of MCL-1 or BCL-X under circumstances where cell death was dependent on c-MYC. However, in SCCRDEB4 cells where SF3B1 knockdown-induced cell death was c-MYC-independent, full-length MCL-1 protein expression was reduced by SF3B1 depletion (Figure 3B). Similarly, c-MYC knockdown reduced the sensitivity of SCCRDEBMet and SCCRDEB4 lines to death induced by the SF3B1 inhibitor pladienolide B (Figure 4A). Full-length MCL-1 and BCL-X protein levels were not altered at concentrations of pladienolide B that selectively kill cSCC cells (Figure 4B). However, full-length MCL-1 protein expression was reduced and the expression of low molecular weight MCL-1 isoforms was increased at relatively high concentrations of pladienolide B at which cell death was unaffected by c-MYC knockdown. In cSCC cells endogenous c-MYC can thus confer sensitivity to interference with the spliceosome. Strong suppression of SF3B1 can however kill cSCC cells independently of c-MYC. Altered MCL-1 expression provides a marker of strong suppression of SF3B1 and could contribute to c-MYC-independent cell death. However, changes in MCL-1 expression are not involved in killing cSCC cells at low tumour-selective concentrations of pladienolide B.

**Figure 5:**
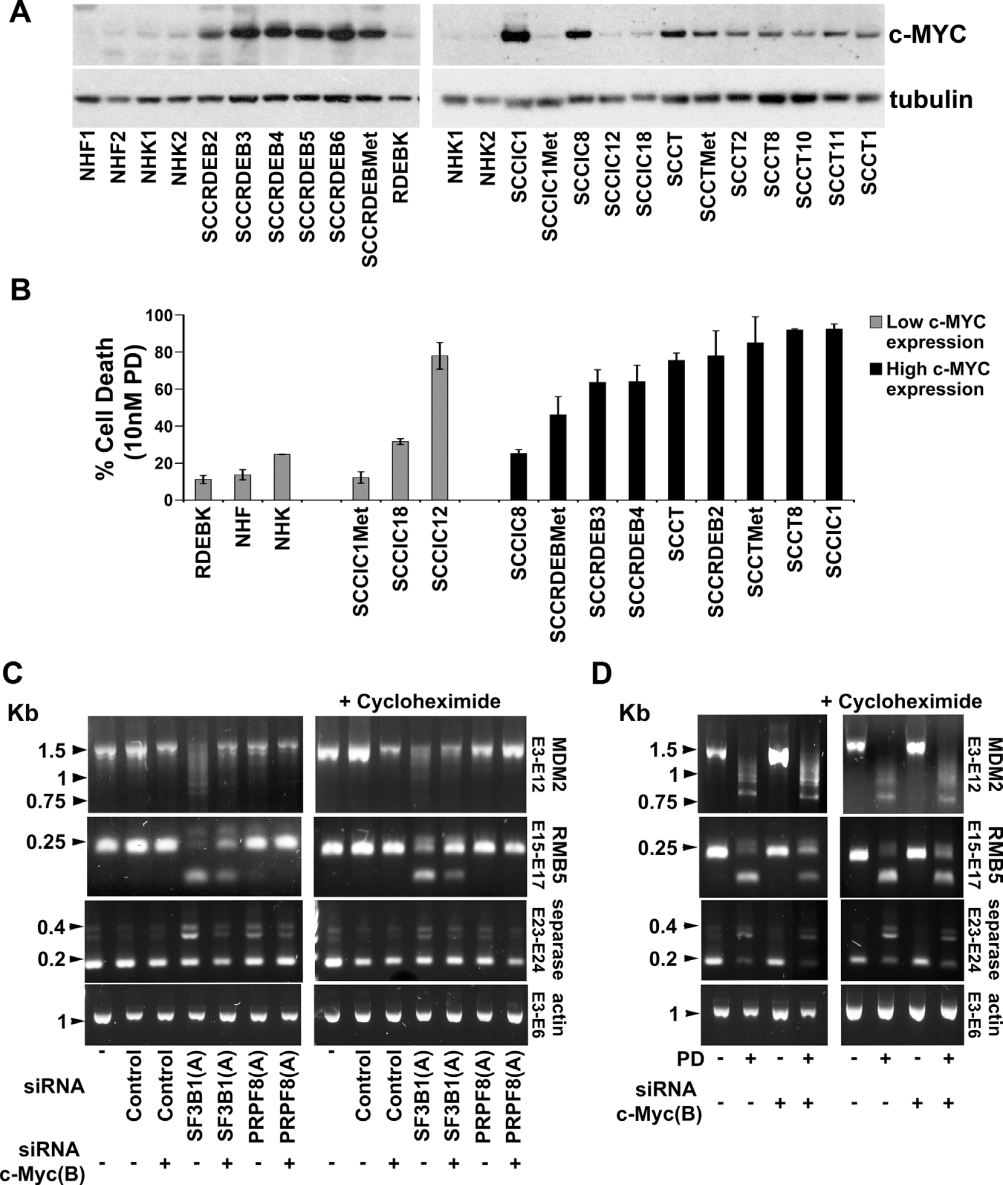
c-MYC is upregulated in cSCC cell lines and promotes altered splicing following interference with the spliceosome (**A**) c-MYC protein expression in normal skin cells (NHF, NHK and RDEBK) and a panel of cSCC cell lines was analysed 72 hours after plating. NHF1 and 2 and NHK1 and 2 were from different donors. c-MYC protein expression was higher in cSCC cells compared to normal cells with the exception of SCCIC1Met, SCCIC12 and SCCIC18 cell lines. (**B**) Cell death at 10 nM pladienolide B (taken from Figure 2). Normal cells and two of the three cSCC cell lines with low c-MYC expression were relatively resistant to pladienolide B. (**C**) SCCRDEBMet cells were transfected with the indicated combinations of siRNAs. RNA was extracted 48 hours after transfection. One set of cells were treated with cycloheximide (20 μg/ml) 6 hours before harvesting to inhibit NMD (right panel). PCR was carried out using primers complementary to the indicated exons (E). (**D**) SCCRDEBMet cells were transfected with non-targeting siRNA (Control) or siRNA c-MYC (B) 24 hours before the addition of 10 nM pladienolide B (PD). One set of cells was treated with cycloheximide 6 hours before the end of the incubation (right panel). Samples were harvested for RNA extraction 24 hours after the initiation of pladienolide B treatment. c-MYC knockdown partially reversed alterations in mRNA splicing caused by interference with the spliceosome.

Consistent with previous results c-MYC protein levels were upregulated compared to normal skin cells in fifteen of the eighteen cSCC cell lines analysed which provides a mechanism for the cSCC selectivity of interference with the spliceosome (Figure 5A) [19, 38, 64, 65]. Consistent with this, two of the three cSCC cell lines (SCCIC1Met and SCCIC18) with low c-MYC expression were relatively insensitive to pladienolide B (Figures 2 and 5B). We also observed that PRPF8 and SF3B1 were generally upregulated in cSCC cells (Supplementary Figure 2). This may be due to an increased demand for splicing in the tumour cells, however there was no association between levels of these splicing factors and the differential pladienolide B sensitivity of cSCC cell lines.

The effect of c-MYC knockdown on changes in splicing caused by suppression of SF3B1 and PRPF8 was investigated. Selected splicing events known to be dependent on PRPF8 and/or SF3B1 were assessed (Figure 5C) [49, 66, 67]. SF3B1 depletion resulted in exon skipping in MDM2 and RBM5 mRNAs and intron retention in separase mRNA. PRPF8 depletion caused an increase in separase mRNA intron retention. c-MYC knockdown reduced the severity of these alterations in splicing. The effect of pladienolide B on the splicing events examined was similar to that of SF3B1 knockdown. Depletion of c-MYC attenuated the changes in the splicing of RBM5 and separase mRNAs caused by pladienolide B (Figure 5D). Alternatively/aberrantly spliced forms of mRNA can be degraded by the translation coupled nonsense-mediated mRNA decay (NMD) pathway [68, 69]. Similar results were observed in the absence and presence of the translation inhibitor cycloheximide which was added to block degradation of potential NMD substrates. These data indicate that endogenous c-MYC can promote alterations in splicing caused by targeting the spliceosome.

### Wild-type p53 participates in killing normal skin cells following suppression of SF3B1

**Figure 6:**
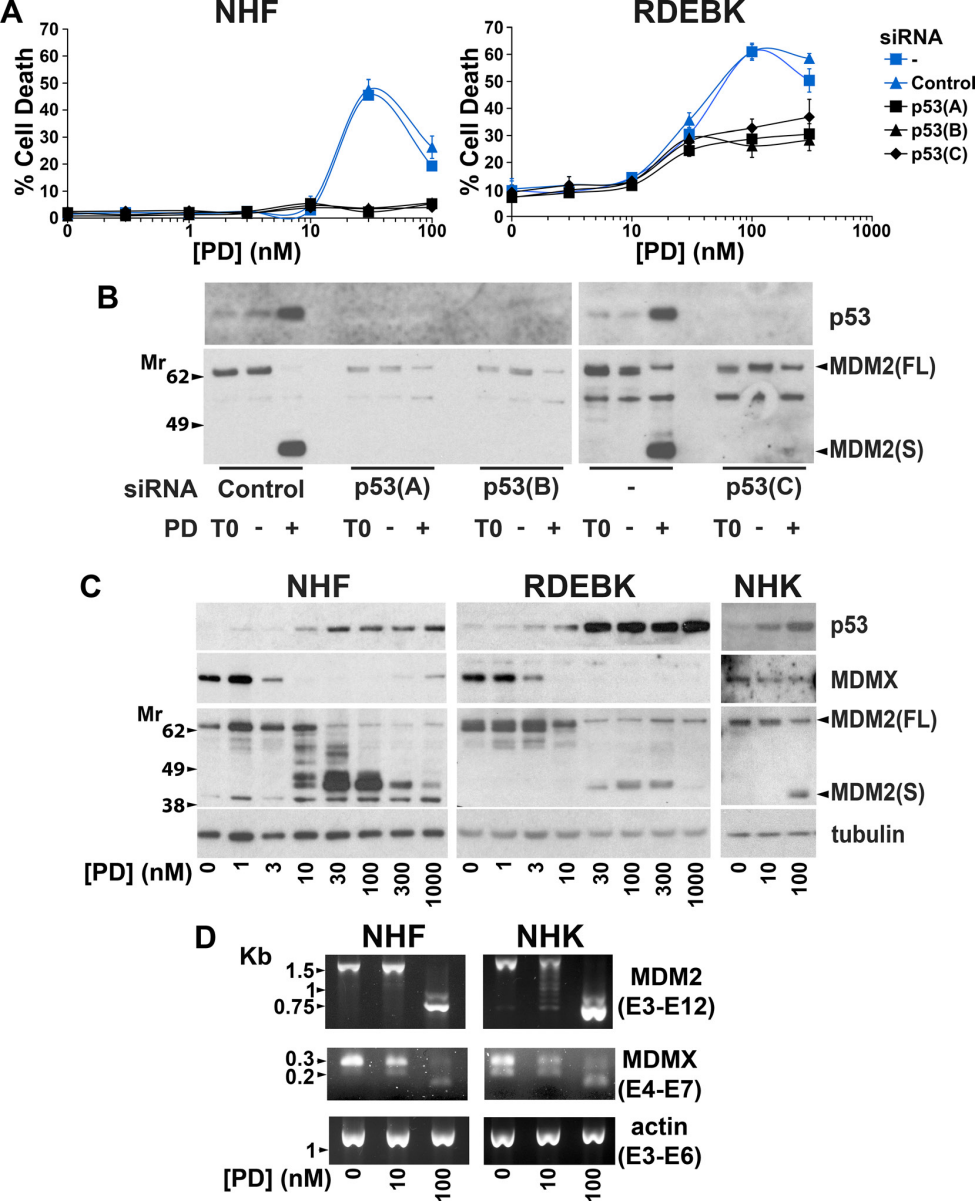
p53 plays a major role in killing normal skin cells following exposure to pladienolide B (**A**) 24 hours prior to the addition of pladienolide B (PD) NHF and RDEBK were transfected with three individual siRNAs targeting all known isoforms of p53. Cell death was analysed by real-time imaging 72 hours after pladienolide B addition. Knockdown of p53 reduced sensitivity to pladienolide B. (**B**) NHF were transfected with three individual p53 siRNAs. 24 hours after transfection cells were treated with carrier (-) or 100 nM pladienolide B (+). Cells were harvested at the time of drug addition (T0) and 24 hours later. p53 was efficiently knocked down by all of the siRNAs. (**C**) NHF, RDEBK and NHK were treated with pladienolide B (PD) for 24 hours. Protein expression was analysed by western blotting. Strong p53 upregulation was associated with reduced full-length MDM2 (MDM2FL) protein expression. (**D**) NHF and NHK cells were treated with pladienolide B (PD) for 24 hours. PCR was carried out with primers complementary to the indicated exons (E). 10 and 100 nM pladienolide B altered the ratio of alternatively spliced forms of MDMX mRNA and 100 nM pladienolide B dramatically interfered with MDM2 mRNA splicing.

We previously observed that the p53 pathway is involved in sensing alterations in the spliceosome [49]. The role of p53 in the response of normal skin cells to pladienolide B was investigated to determine if p53 activation may limit the therapeutic window for SF3B1 inhibition. Knockdown of p53 reduced pladienolide B-dependent death in both NHF and RDEBK cells (Figure 6A and 6B). In normal skin cells protein expression of the p53 repressor MDMX was reduced at low concentrations of pladienolide B, associated with changes in the ratio of alternatively spliced forms of MDMX mRNA (Figure 6C and 6D). This was not sufficient to cause marked p53 accumulation or cell death. The pladienolide B concentration dependency for killing normal skin cells corresponded to that for causing: altered MDM2 mRNA splicing, a reduction in full-length MDM2 protein expression and a robust increase in p53 protein levels (Figures 2 and 6). These results indicate that induction of wild-type p53 due to loss of full-length MDM2, in the context of reduced MDMX expression, plays an important role in killing normal skin cells in response to pladienolide B.

Normal p53 function is lost, most commonly by its missense mutation, in all cSCC cell lines [64]. We confirmed that knockdown of mutant p53 in cSCC cells did not attenuate pladienolide B-induced death (Supplementary Figure 3). Pladienolide B and SF3B1 knockdown reduced MDMX and MDM2 protein expression in cSCC lines, indicating that these effects can occur independently of wild-type p53 (Supplementary Figures 4 and 5). Low molecular weight protein isoforms of MDM2 were detected in cells with wild-type p53 following treatment with pladienolide B but not in cSCC cells with mutant p53 (Figure 6 and Supplementary Figure 4). Consistent with this, pladienolide B-dependent expression of short MDM2 protein isoforms was attenuated by knockdown of wild-type p53 in NHF and loss of full-length wild-type p53 in HCT116 cells (Figure 6B and Supplementary Figure 6B). The mechanism underlying the p53-dependent accumulation of MDM2 isoforms remains unclear but it could involve elevated *MDM2* gene transcription due to upregulation of wild-type p53 [49]. SF3B1 suppression did not increase levels of mutant p53 in cSCC cell lines (Supplementary Figures 4A and 5A). This is consistent with the high protein stability of mutant p53 in tumours [70]. On the contrary, we observed that 3 to 30 nM pladienolide B and SF3B1 knockdown actually decreased mutant p53 protein expression to some extent in cSCC cells. This is unlikely to make a major contribution to death caused by SF3B1 suppression through attenuation of mutant p53 gain of function activity because p53 could be robustly knocked down in cSCC cell lines without causing marked cell death (Supplementary Figure 3).

**Figure 7:**
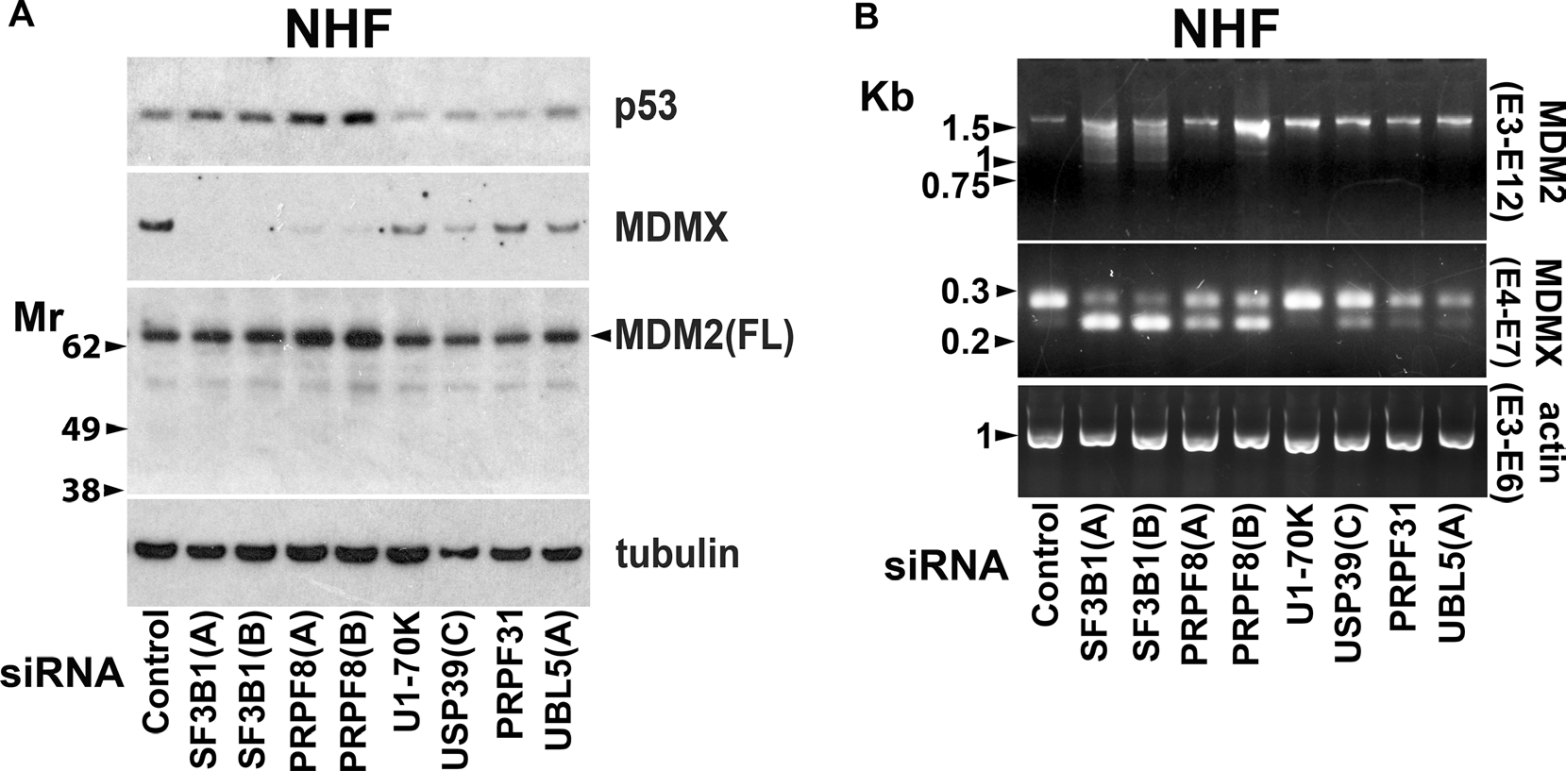
Altered MDM2 mRNA splicing is selectively caused by suppression of SF3B1 NHF were transfected with siRNAs targeting the indicated splicing factors. Samples were analysed 48 hours after transfection. (**A**) Western blot analysis. Splicing factor knockdown reduced MDMX protein expression. (**B**) PCR analysis. With the exception of U1-70K, suppression of splicing factors altered the ratio of alternatively spliced forms of MDMX. Only SF3B1 knockdown altered splicing of MDM2 mRNA.

We have previously observed in tumour cells that MDMX protein levels are reduced by suppression of a range of splicing factors but that MDM2 mRNA splicing is selectively sensitive to knockdown of SF3B1 [49]. Consistent with this, PRPF8 knockdown in cSCC cells reduced full-length MDMX protein expression but it did not diminish MDM2 protein levels (Supplementary Figure 5B). Targeting a range of splicing factors, including SF3B1, with previously validated siRNAs reduced MDMX protein levels in NHF (Figure 7A) [49]. In most cases this was associated with an altered ratio of alternatively spliced forms of MDMX mRNA (Figure 7B). In contrast, knockdown of SF3B1 but not the other splicing factors disrupted MDM2 mRNA splicing (Figure 7B). Depletion of at least some of these splicing factors can kill cSCC cells (Figure 1). This indicates that targeting splicing factors other than SF3B1 could have anti-cSCC activity without altering MDM2 mRNA splicing in normal cells. This would be advantageous because loss of MDMX alone has less impact on normal tissues than loss of both MDMX and MDM2 [51, 71–73]. SF3B1 knockdown altered MDM2 splicing in NHF but this did not change MDM2 protein expression and p53 levels were not strongly upregulated by SF3B1 knockdown (Figure 7A). This presumably reflects partial depletion of SF3B1 and is consistent with the relatively low level of death induced by SF3B1 knockdown in NHF and a requirement for stronger suppression of the spliceosome to have effects on splicing in normal skin cells. At high concentrations pladienolide B was able to suppress SF3B1 sufficiently to reduce full-length MDM2 protein expression in normal cells (Figure 6).

To begin to investigate the involvement of endogenous wild-type p53 in killing tumour cells in response to SF3B1 inhibition the effects of pladienolide B were compared in HCT116 (p53+/+) colon cancer cells and a derivative cell line lacking full-length p53 (p53-/−). The absence of full-length p53 markedly reduced cell death caused by pladienolide B (Supplementary Figure 6A). HCT116 (p53+/+) cells were killed at lower concentrations of pladienolide B than normal cells (Supplementary Figure 6A and Figures 2 and 6A). A reduction in MDM2 and MDMX protein expression and accumulation of p53 also occurred at lower concentrations of pladienolide B in HCT116 cells than in normal cells (Supplementary Figure 6B and Figure 6C). These results indicate that wild-type p53 upregulation associated with downregulation of MDM2 and MDMX can also play a dominant role in the killing of tumour cells by SF3B1 inhibition and that there may be a level of tumour selectivity because of the greater sensitivity of MDM2/MDMX expression in transformed cells.

## DISCUSSION

This study indicates that targeting the spliceosome has potential for the treatment of cSCC. We show that endogenous c-MYC participates in sensitising cSCC cells to interference with the spliceosome. We provide evidence for a role of the p53 pathway in determining the therapeutic window for SF3B1 suppression.

Small molecule inhibition of SF3B1 can selectively kill cSCC cells. There is an ongoing clinical trial for haematological malignancies involving systemic delivery of an SF3B1 inhibitor and additional SF3B inhibitors are candidates for testing in the clinic [28]. cSCC cell lines can also be more sensitive than normal skin cells to death induced by suppression of splicing factors other than SF3B1. This suggests that SF3B1-independent targeting of the spliceosome could also be effective for treating cSCCs. There are variations between cSCC cell lines in the potency of targeting different splicing factors. This may be due to depletion of different components of the spliceosome influencing distinct subsets of splicing events [22]. Divergent downstream mechanisms could mediate the anti-tumour activity of different ways of targeting the spliceosome. There are a number of mechanisms through which interference with the spliceosome and altered pre-mRNA splicing could cause cell death including: reduced expression of components or regulators of key cellular pathways, the inappropriate expression of protein isoforms that interfere with specific cellular pathways and the generation of aberrant misfolded proteins which trigger protective mechanisms such as the unfolded protein response [74]. It would be of interest to identify the downstream mechanisms involved in killing cSCC cells following the suppression of different components of the spliceosome. This would aid in determining how to target the spliceosome for cSCC therapy and may lead to the discovery of additional makers of sensitivity and potential combinatorial therapeutic approaches.

Our study indicates that c-MYC upregulation contributes to the cSCC selectivity of interference with the spliceosome. This could be due at least in part to its ability to increase the severity of alterations in splicing caused by targeting the spliceosome. An implication of this is that irrespectively of whether different ways of targeting the spliceosome have anti-tumour activity through divergent downstream mechanisms c-MYC could act as a common sensitizer by increasing the extent of alterations in splicing. These results are consistent with a model in which high levels of c-MYC stresses the spliceosome by increasing the demand for splicing through widespread upregulation of transcription [19]. Elevated c-MYC can cause an increase in total cellular pre-mRNA/mRNA levels through direct transcriptional activation of genes and/or by more indirect mechanisms [19, 75–77]. c-MYC protein expression is high in around 80% of patient cSCCs [39, 40, 78]. This could provide a marker for sensitivity to spliceosome inhibition, although unsurprisingly our data indicate that other markers would also be required. This might include markers of c-MYC transcriptional activity or of additional mechanisms of sensitivity/resistance.

The p53 pathway is responsive to interference with the spliceosome. This could reflect a normal role in sensing and protecting against potentially tumour promoting alterations in the spliceosome. There are often widespread/global changes in splicing during tumour development which can be linked with aberrant expression of splicing factors and to splicing factor mutations [79–84]. SF3B1 is the most frequently mutated splicing factor in tumours. The spliceosome is also involved in preventing DNA damage, ensuring proper mitosis and maintaining genome stability [85–87]. We observed that knockdown of all splicing factors tested reduces MDMX protein expression in normal skin cells, including splicing factors outwith the SF3B complex that are associated with different snRNPs and splicing factors involved in the formation of multi-snRNP complexes. This is consistent with our previous observations and is further evidence that MDMX may act as a relatively general sensor of perturbations in the spliceosome [49]. Of the splicing factors that we have targeted to date only SF3B1 knockdown results in an alteration in MDM2 splicing. The convergence on SF3B1 is intriguing, in that: MDM2 splicing appears to be particularly sensitive to SF3B1 suppression, SF3B1 is the target for multiple families of naturally occurring splicing modulators and there is an elevated SF3B1 mutation rate in cancer. This is despite the multitude of splicing factors with important roles in splicing.

Death of normal skin cells at high concentrations of pladienolide B is p53-dependent and is associated with altered mRNA splicing and reduced protein expression of both MDMX and MDM2. MDMX is reduced at lower pladienolide B concentrations than MDM2. However, this reduction in MDMX is not sufficient to cause strong p53 accumulation or cell death. This is consistent with the severity of p53-dependent effects on normal tissues in mouse knockout models in which loss of MDM2 is more deleterious than loss of MDMX and targeting MDMX and MDM2 simultaneously has the greatest impact [51, 71–73]. It is also consistent with observations that the effects of MDMX suppression on p53 protein levels are generally smaller than those of targeting MDM2 [51, 52].

p53 function is commonly lost through mutation in cSCC [64, 88–90]. We confirmed that missense mutant p53 depletion did not attenuate pladienolide B-induced cell death in cSCC cells. Knockdown of splicing factors outwith the SF3B complex can kill cSCC cells expressing mutant p53 without altering MDM2 splicing. This raises the possibility that by not depleting both MDM2 and MDMX and thus avoiding strong p53 upregulation in normal cells small-molecules that target the spliceosome through SF3B1-independent mechanisms could have even greater selectivity than SF3B1 inhibitors for cSCCs with mutant p53. Furthermore, it may be advantageous to treat cSCCs where p53 is mutated with an SF3B1-independent inhibitor because a reduction in MDMX and MDM2 levels could enhance the gain of function activity of mutant p53. Although not the case in the cSCC lines examined, MDM2 depletion in some contexts can increase mutant p53 expression [91]. In addition, MDM2 and MDMX have been reported to suppress at least some gain of function activities of mutant p53 [92, 93].

As p53 is mutated in all existing cSCC lines we used HCT116 colon cancer cells to explore the role of wild-type p53 in the response of tumours cells to SF3B1 inhibition. These cells undergo p53-dependent cell death at lower pladienolide B concentrations than the normal cells examined. This indicates that wild-type p53 can contribute to the selective killing of tumour cells. Intriguingly, full-length MDMX and MDM2 protein expression was reduced at lower concentrations of pladienolide B in HCT116 cells than in the normal cells. A number of agents have been developed for treating cancers with wild-type p53 that target MDMX and MDM2 alone or in combination [94–96]. The majority of these prevent the interaction of MDMX and/or MDM2 with p53. However, there are also agents with different mechanisms of action including inhibitors of MDMX/MDM2 heterodimerisation and antisense oligonucleotides that promote MDMX exon skipping. Although not specific for the p53 pathway, modulation of SF3B1 is another way of suppressing MDMX and MDM2. Targeting the spliceosome by SF3B1-independent mechanisms also provides a potential therapeutic approach for reducing MDMX expression. This study indicates that there may be a window for tumour-selective depletion of MDMX/MDM2 by spliceosome inhibition. Additional work is required to identify the optimum ways of targeting the spliceosome to activate wild-type p53 in tumour cells with the least potential for adverse effects in normal tissue.

It would be of interest in further studies to compare the anti-tumour activity and selectivity of SF3B1-independent small molecule suppressors of the spliceosome and to investigate the roles of c-MYC, the p53 pathway and their cross-talk in determining responses. Inhibitors which directly target components of the spliceosome other than SF3B1 are being developed [97–100]. There are also inhibitors of kinases that play important roles in regulating the spliceosome [101]. In addition, the therapeutic potential of targeting the spliceosome could be further mapped by the systematic knockdown of all core splicing factors and key spliceosome regulators in normal cells and tumour cells with different p53 status and determination of the effect on cell viability/death, MDM2, MDMX and p53. This would contribute to the identification of the best approaches for targeting the spliceosome for cancer therapy.

## MATERIALS AND METHODS

### Cell culture

Cells were cultured at 37°C and 5% CO_2_ in a humidified atmosphere. Normal keratinocytes (NHK and RDEBK) and cSCC cells were isolated and maintained as described [64, 102]. These cells were cultured in keratinocyte medium containing 10% FBS and growth factors [102]. Normal keratinocytes were cultured in the presence of a mitotically inactivated 3T3 fibroblast feeder layer. RDEBK were routinely expanded using the ROCK inhibitor Y-27632 (1254: Tocris Bioscience, Bristol, UK) and this was removed 48 hours before the start of experiments [103]. SCCT (MET1) and SCCTMet (MET4) cell lines were described previously [104]. SCCRDEB4 and SCCRDEBMet (SCCRDEB70) cell lines were from different patients. SCCRDEBMet and RDEBK cells were a gift from Dr Andrew P. South (Thomas Jefferson University). The tissue from which these cells were derived was provided by Jemima E. Mellerio (King’s College London) and Julio C. Salas-Alanís (DEBRA Mexico) [65]. SCCT/SCCTMet and SCCIC1/SCCIC1Met cell lines are from paired primary and metastatic tumours. NHF were isolated as described and routinely cultured in Dulbecco’s Modified Eagle Medium (419660-29: Gibco, Thermo Fisher Scientific, Waltham, MA USA) containing 10% FBS [105]. For experiments, normal skin cells and cSCC lines were plated in the absence of feeders in keratinocyte medium containing 10% serum and growth factors with no added EGF. HCT116 cells were cultured and plated in McCoy’s 5A medium (26600-023: Gibco, Thermo Fisher Scientific), supplemented with 10% FBS.

### Pladienolide B treatment

Pladienolide B (sc-391691: Santa Cruz Biotechnology, Heidelberg, Germany) was dissolved in DMSO (5 mM stock) and was added to cells 16 to 24 hours after plating/transfection with siRNAs unless otherwise indicated.

### siRNA transfection

Dharmacon ON-TARGETplus modified siRNAs (Thermo Fisher Scientific) were used (Supplementary Figure 7A) with the exception of siRNA p53 (C) which is described previously [106]. Reverse transfection with synthetic siRNA duplexes (10 nM final concentration) was carried out using Invitrogen Lipofectamine RNAiMAX transfection reagent (13778150: Thermo Fisher Scientific) according to the manufacturer’s instructions.

### Cell viability assays

Cells were seeded into 96 well plates and cell viability (live cell number) and cell death were analysed according to the manufacturers’ instructions using an Incucyte ZOOM real-time imager (Essen BioScience Ltd, Welwyn Garden City, UK) and the CellTox Green cytotoxicity assay (G8731: Promega, Southampton, UK). Live cell number and cell death were generally analysed 96 hours after transfection with siRNAs and/or 72 hours after pladienolide B addition. This was extended to 120 and 96 hours respectively for experiments investigating the effect of c-MYC and p53 knockdown in SCCRDEBMet cells.

### Western blotting

Cell extracts were made by lysis into SDS electrophoresis sample buffer: 100 mM Tris pH 6.8, 4% SDS, 20% glycerol, 20 mM EDTA, 0.014% bromophenol blue. Western blotting was performed as described previously [107]. The primary antibodies used are listed in Supplementary Figure 7B.

### RNA preparation and PCR

Where indicated cells were treated with cycloheximide (20 μg/ml) for 6 hours before harvesting to block degradation of potential NMD substrates. Total RNA was extracted using RNeasy columns (Qiagen, Crawley, UK). 1 μg of RNA was reverse-transcribed using random primers (58875: Invitrogen, Thermo Fisher Scientific). The cDNA (5% of the reverse-transcription reaction) was amplified by PCR using the primers listed in Supplementary Figure 7C. After 40 cycles of 1 min at 95°C, 45 s at 56°C and 1 min at 72°C, the products were analysed on agarose gels.

## SUPPLEMENTARY MATERIALS FIGURES



## Abbreviations

(PD): Pladienolide B
(cSCC): cutaneous squamous cell carcinoma
(NHF): normal human fibroblasts
(NHK): normal human keratinocytes
(NMD): nonsense-mediated mRNA decay
(RDEB): recessive dystrophic epidermolysis bullosa
(RDEBK): recessive dystrophic epidermolysis bullosa keratinocytes
(SCCIC): squamous cell carcinoma immunocompetent
(SCCRDEB): squamous cell carcinoma recessive dystrophic epidermolysis bullosa
(SCCT): squamous cell carcinoma transplant
(snRNA): small nuclear RNA
(snRNP): small nuclear ribonucleoprotein
