# Supplementary material for: Targeting the spliceosome for cutaneous squamous cell carcinoma therapy: a role for c-MYC and wild-type p53 in determining the degree of tumour selectivity
Source: Oncotarget. 2018 May 1;9(33):23029–46. doi: 10.18632/oncotarget.25196 (PMC5955416; doi:10.18632/oncotarget.25196)
Supplement: Supplementary file 1 [file oncotarget-09-23029-s001.pdf]

Targeting the spliceosome for cutaneous squamous cell carcinoma therapy: a role for c-MYC and wild-type p53 in determining the degree of tumour selectivity

SUPPLEMENTARY MATERIALS

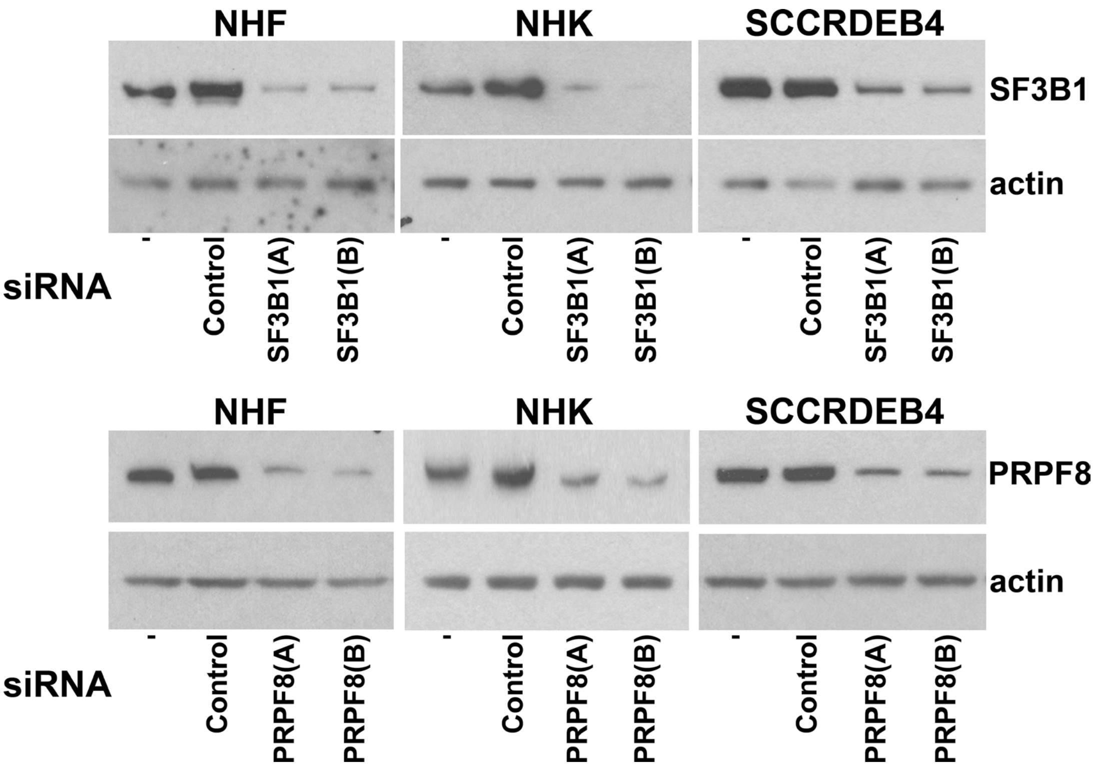

Supplementary Figure 1: Splicing factors are efficiently knocked down in both normal skin cells and cSCC cells. NHF, NHK and SCCRDEB4 cells were mock transfected (−) or transfected with the indicated siRNAs. SF3B1 and PRPF8 knockdown was assessed by western blotting 48 hours after transfection.

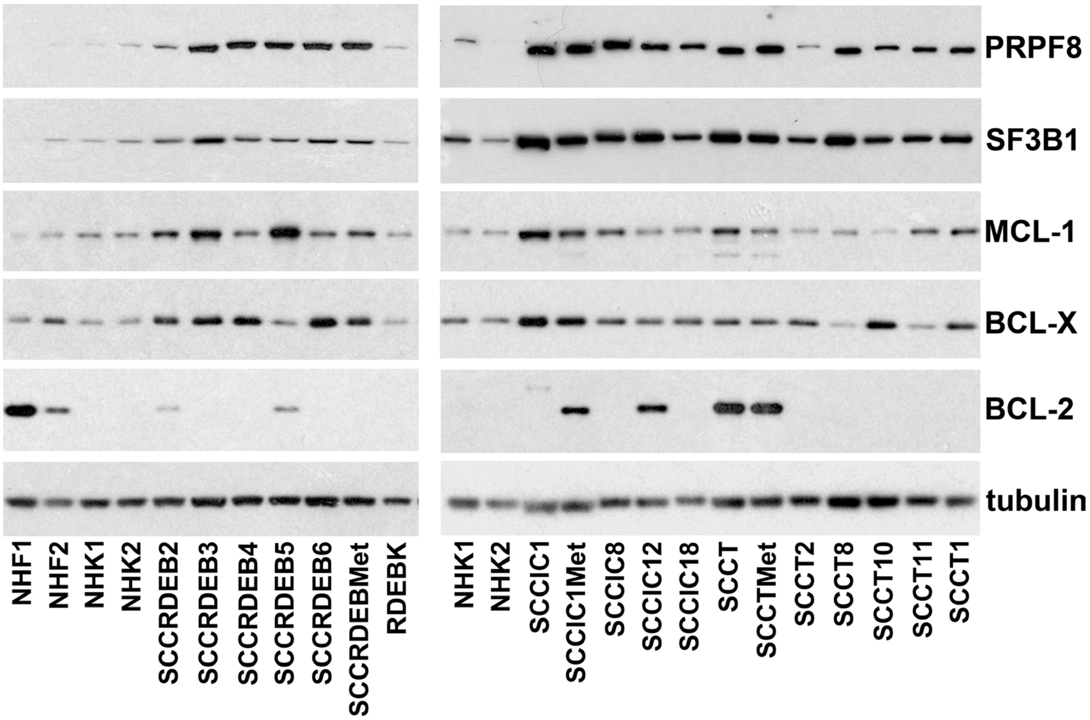

Supplementary Figure 2: Protein expression of PRPF8, SF3B1 and BCL-2 family members in cSCC cell lines. Protein expression was analysed 72 hours after plating normal skin cells (NHF, NHK and RDEBK) and cSCC cell lines. NHF1 and 2 and NHK1 and 2 were from different donors. The splicing factors SF3B1 and PRPF8 were generally upregulated in cSCC cell lines compared to normal skin cells. All cSCC cell lines expressed MCL-1 and BCL-X.

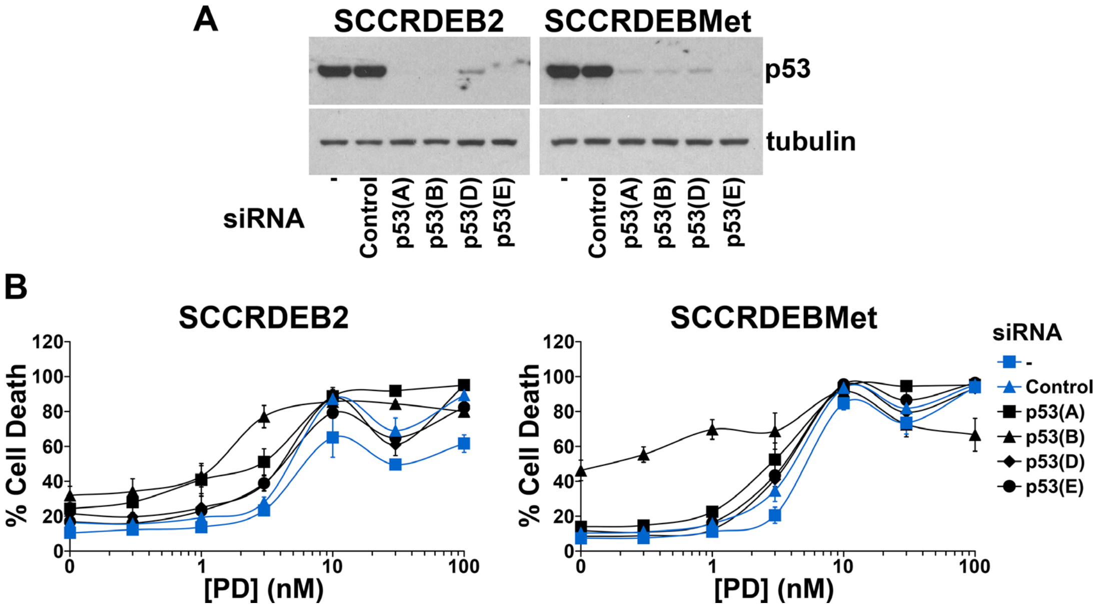

Supplementary Figure 3: Mutant p53 knockdown does not inhibit pladienolide B-induced cell death in cSCC cell lines. (**A**) Mutant p53 protein expression was reduced within 48 hours of transfection of cSCC cells with four individual p53 siRNAs. (**B**) cSCC cells were treated with pladienolide B (PD) 48 hours after transfection with siRNAs targeting p53. Cell death was analysed by real-time imaging. Values are the mean −/+ SEM of three independent experiments. With three of the four siRNAs there was little or no effect on pladienolide B sensitivity. siRNA p53 (A) caused a high basal level of cell death particularly in SCCRDEBMet cells. This was most likely due to context-dependent off target effects.

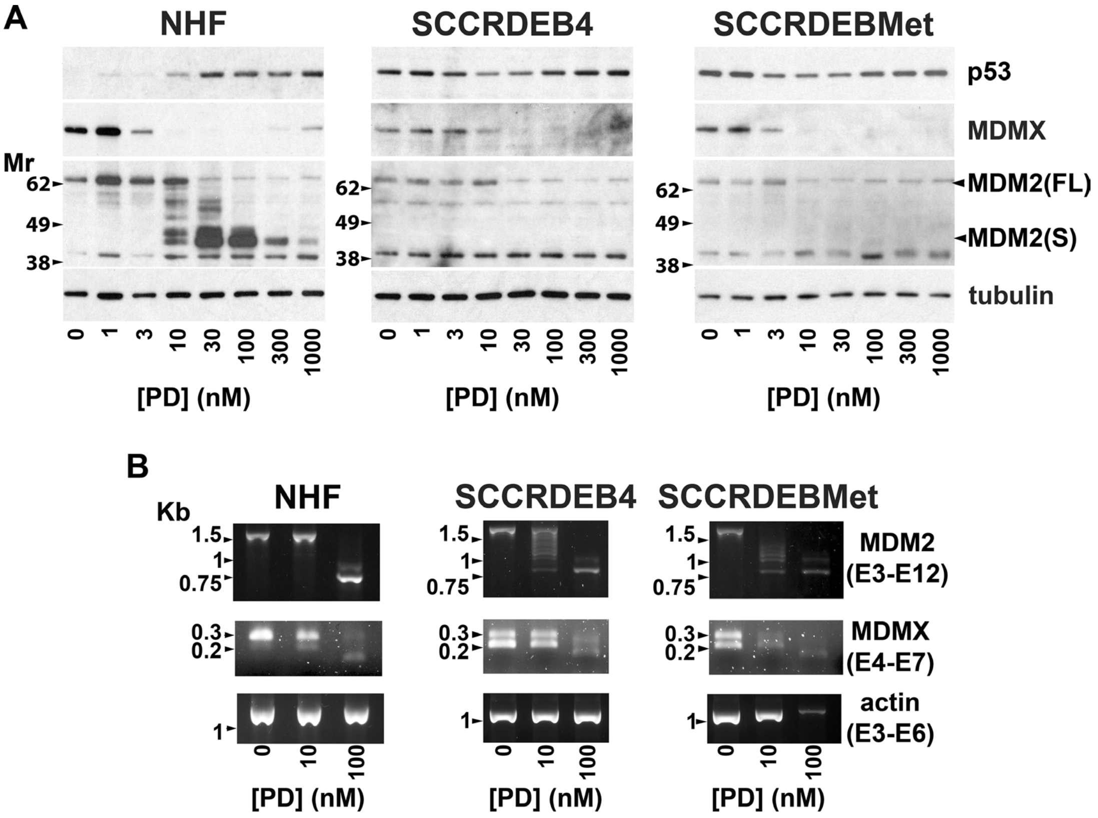

Supplementary Figure 4: Pladienolide B treatment of cSCC cell lines reduces MDM2 and MDMX protein expression but does not cause the accumulation of mutant p53. Mutant p53 expressing SCCRDEB4 and SCCRDEBMet cells were treated with pladienolide B for 24 hours. The panel showing the effect on NHF treated in an identical manner is taken from Figure 6C and is included here for comparison. (**A**) Protein expression was assessed by western blotting. In cSCC cells pladienolide B reduced full-length MDMX and MDM2 but p53 and short MDM2 isoforms (MDM2S) were not accumulated. (**B**) Splicing of MDM2, and MDMX was assessed by PCR using primers complementary to the indicate exons (E). Splicing of MDMX and MDM2 mRNA was altered by pladienolide B in cSCC cells.

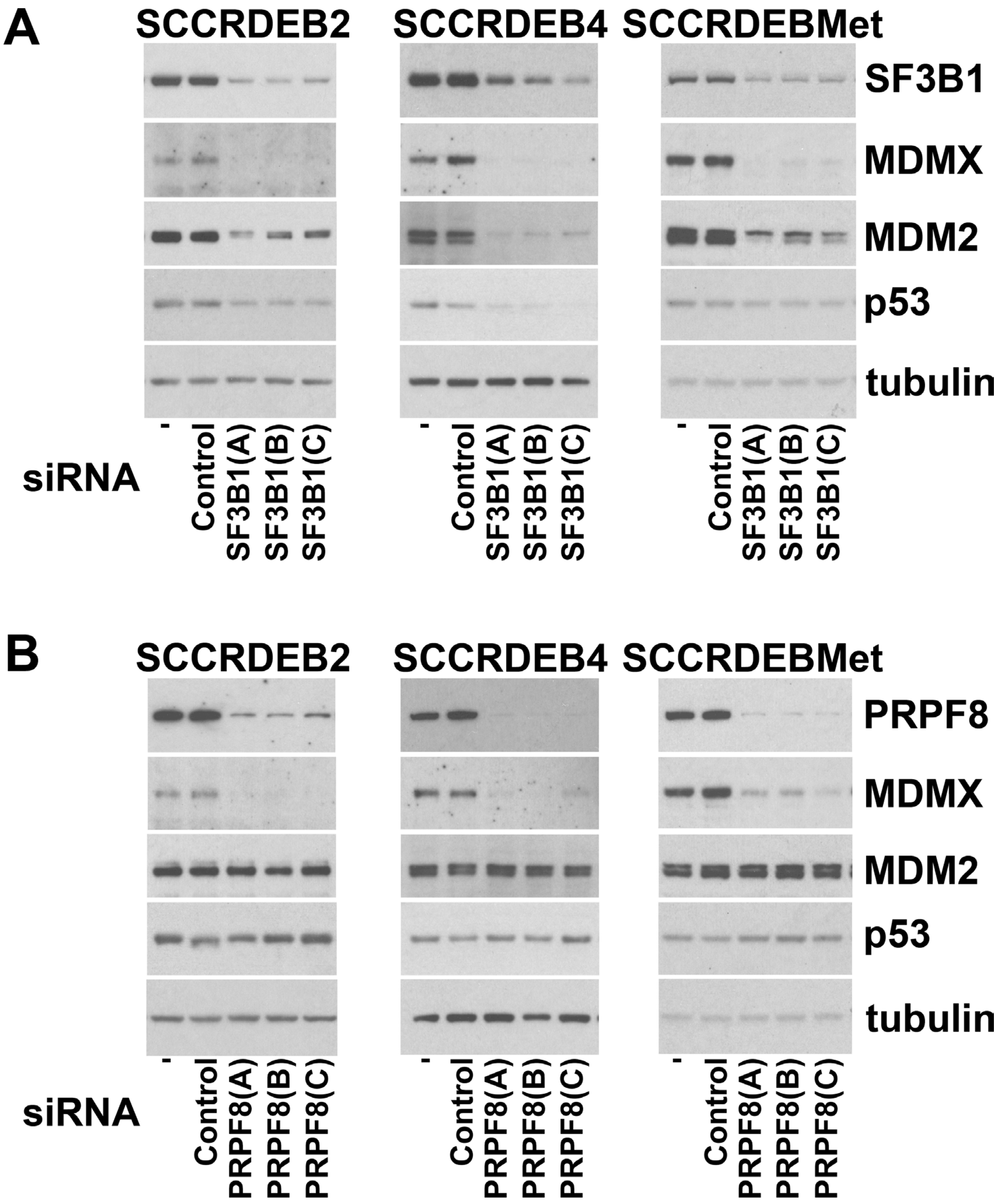

Supplementary Figure 5: SF3B1 and PRPF8 knockdown in cSCC cell lines has a differential effect on MDM2 and p53 protein levels. cSCC cell lines expressing mutant p53 were transfected with the indicated siRNAs and protein expression was analysed by western blotting after 48 hours. (**A**) SF3B1 knockdown reduced full-length MDMX, MDM2 and p53 expression. (**B**) PRPF8 knockdown reduced MDMX levels but it did not alter MDM2 and p53 expression.

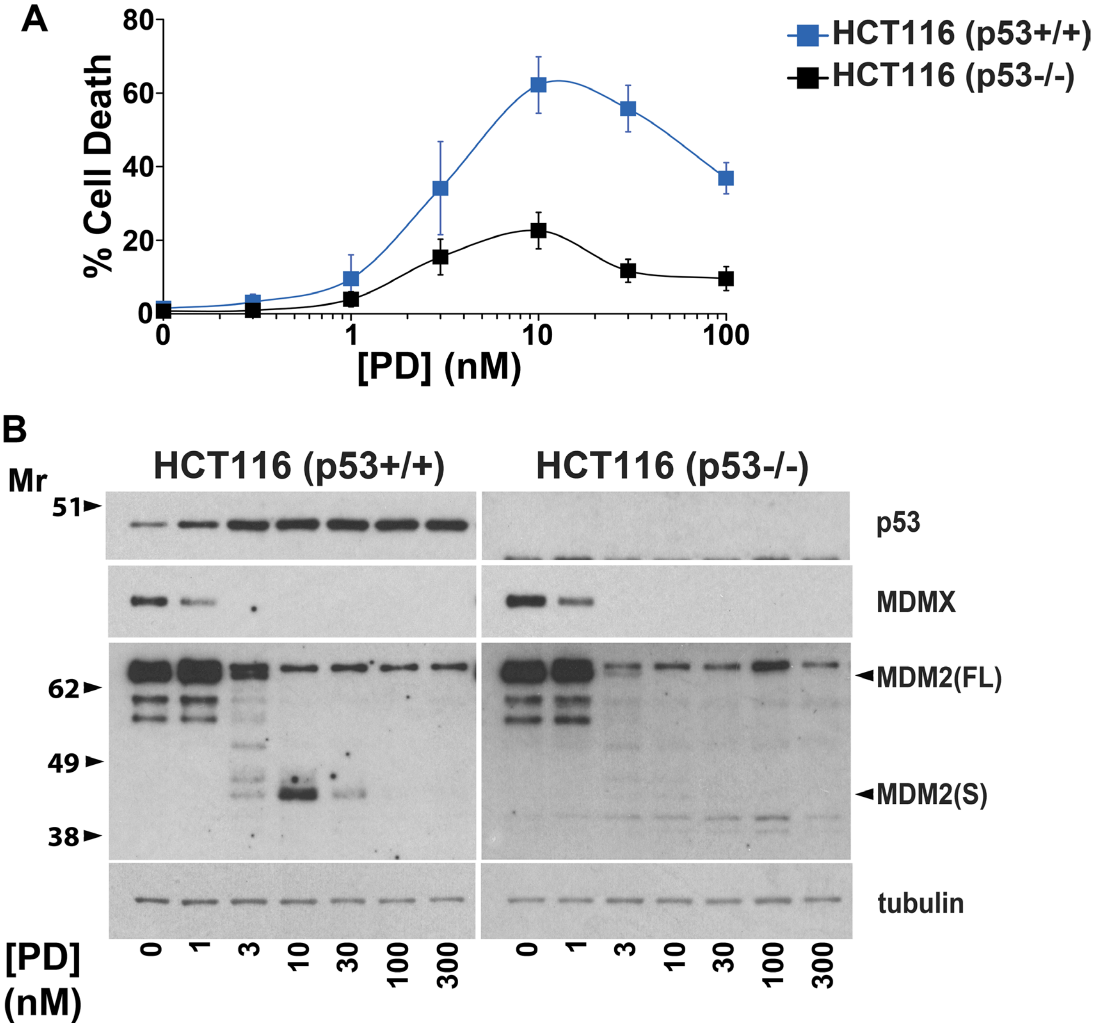

Supplementary Figure 6: Pladienolide B-induced cell death is p53-dependent in HCT116 colon cancer cells. (**A**) HCT116 cells with endogenous full-length wild-type p53 (p53+/+) or a derivative where full length p53 expression is abolished (p53−/−) were incubated with pladienolide B (PD) for 72 hours and cell death was analysed by real-time imaging. Values are the mean –/+ SEM of three independent experiments. Loss of full-length p53 reduces pladienolide B-induced cell death. (**B**) HCT116 cells were incubated with pladienolide B (PD) for 24 hours. Protein expression was analysed by western blotting. Low concentrations of pladienolide B reduced full-length MDMX and MDM2 protein expression in both HCT116 (p53+/+) and HCT116 (p53−/−) cells.

A siRNAs (Dharmacon ON-TARGETplus):

Control: D-001810-01.
c-MYC(A): J-003282-25, c-MYC(B): J-003282-26.
p53(A): J-003329-14, p53(B) J-003329-17, p53(D): J-003329-15, p53(E): J-003329-16
PHF5A(A): J-014987-09, PHF5A(B): J-014987-10, PHF5A(C): J-014987-11.
PRPF8(A): J-012252-06, PRPF8(B): J-012252-07, PRPF8(C): J-012252-09.
PRPF19(A): J-004668-05, PRPF19(B): J-004668-06, PRPF19(C): J-004668-07.
PRPF31: J-020525-05.
SF3B1(A): J-020061-13 , SF3B1(B): J-020061-14, SF3B1(C): J-020061-16.
SF3B3(A): J-020085-05, SF3B3(B): J-020085-06, SF3B3(C): J-020085-08.
Tox: D-001500-01.
U1-70K: J-018811-06.
UBL5(A): J-014320-06, UBL5(B): J-014320-07, UBL5(C): J-014320-08.
USP39(A): J-006087-06, USP39(B): J-006087-07, USP39(C): J-006087-08.

B Primary Antibodies:

β-Actin (ab8226: Abcam, Cambridge, UK).
BCL-2 50E3 (2870P: Cell Signaling Technology, Danvers, MA, USA).
BCL-X 54H6 (2764P: Cell Signaling Technology).
MDM2 2A10 (Prepared in house).
MDMX 8C6 (04-1555: Merck, Watford, UK).
MCL-1 D35A5 (5453P: Cell Signaling Technology).
c-MYC 9E10 (Prepared in house).
p53 SAPU (Scottish Antibody Production Unit, Carluke, UK).
PRPF8 F-6 (sc-55534: Santa Cruz Biotechnology, Heidelberg, Germany).
SF3B1 (D138-3: MBL International, Des Plaines, IL, USA).
α-Tubulin DM1A (T9026: Sigma-Aldrich, Dorset, UK).

C Primers for PCR:

Actin forward E3: 5'-CCACACCTTCTACAATGAGC-3'
Actin reverse E6: 5'-ACAATGTGCAATCAAAGTCC-3'
Mdm2 forward E3: 5'-GTACCTACTGATGGTGCTGTAAC-3'
Mdm2 reverse E12: 5'-AGTTAGCACAATCATTTGAATTG-3'
MDMX forward E4: 5'-TTTATGATCAGCAGGAGCAG-3'
MDMX reverse E7: 5'-TGGGGATATCGTCTTCTGTA-3'
RBM5 forward E15: 5'-CGGCTGTAGTGTCCCAGAGT-3'
RBM5 reverse E17: 5'-TTGCGAGTTGGGGTCATAAT-3'
Separase forward E23: 5'-CAACACCCTCCTGCTGACC-3'
Separase reverse E24: 5'-GTCCACCATTCTCGCTTGTC-3'

Supplementary Figure 7: List of materials used in the study. (**A**) Synthetic siRNA duplexes. (**B**) Primary antibodies. (**C**) PCR primers.
